# Therapeutic Potential of Myrtenal and Its Derivatives—A Review

**DOI:** 10.3390/life13102086

**Published:** 2023-10-20

**Authors:** Stela Dragomanova, Velichka Andonova, Konstantin Volcho, Nariman Salakhutdinov, Reni Kalfin, Lyubka Tancheva

**Affiliations:** 1 Department of Pharmacology, Toxicology and Pharmacotherapy, Faculty of Pharmacy, Medical University of Varna, 84 A Tsar Osvoboditel Blvd., 9002 Varna, Bulgaria; stela_dragomanova@abv.bg; 2 Department of Pharmaceutical Technologies, Faculty of Pharmacy, Medical University of Varna, 84 A Tsar Osvoboditel Blvd., 9002 Varna, Bulgaria; velichka.andonova@mu-varna.bg; 3 Department of Medicinal Chemistry, Novosibirsk Institute of Organic Chemistry of the Russian Academy of Sciences, 9 Lavrentiev Av., 630090 Novosibirsk, Russia; volcho@nioch.nsc.ru (K.V.); anvar@nioch.nsc.ru (N.S.); 4 Institute of Neurobiology, Bulgarian Academy of Sciences, Acad. G. Bonchev St., Block 23, 1113 Sofia, Bulgaria; lyubkatancheva@gmail.com; 5 Department of Healthcare, South-West University, 66 Ivan Mihailov St., 2700 Blagoevgrad, Bulgaria

**Keywords:** monoterpene, pharmacophore, chemical modification, antiviral, anticancer, anxiolytic, neuroprotective activity

## Abstract

The investigation of monoterpenes as natural products has gained significant attention in the search for new pharmacological agents due to their ability to exhibit a wide range in biological activities, including antifungal, antibacterial, antioxidant, anticancer, antispasmodic, hypotensive, and vasodilating properties. In vitro and in vivo studies reveal their antidepressant, anxiolytic, and memory-enhancing effects in experimental dementia and Parkinson’s disease. Chemical modification of natural substances by conjugation with various synthetic components is a modern method of obtaining new biologically active compounds. The discovery of new potential drugs among monoterpene derivatives is a progressive avenue within experimental pharmacology, offering a promising approach for the therapy of diverse pathological conditions. Biologically active substances such as monoterpenes, for example, borneol, camphor, geraniol, pinene, and thymol, are used to synthesize compounds with analgesic, anti-inflammatory, anticonvulsive, antidepressant, anti-Alzheimer’s, antiparkinsonian, antiviral and antibacterial (antituberculosis) properties. Myrtenal is a perspective monoterpenoid with therapeutic potential in various fields of medicine. Its chemical modifications often lead to new or more pronounced biological effects. As an example, the conjugation of myrtenal with the established pharmacophore adamantane enables the augmentation of several of its pivotal properties. Myrtenal–adamantane derivatives exhibited a variety of beneficial characteristics, such as antimicrobial, antifungal, antiviral, anticancer, anxiolytic, and neuroprotective properties, which are worth examining in more detail and at length.

## 1. Introduction

Essential oils have been used in traditional medicine to treat various diseases. Before research into their health benefits had begun, they were also used for perfumes and fragrances for many years. Some of their components possess rich biological activity [1,2,3,4,5,6,7] and are used as pharmacological agents such as the taxanes for their antitumor activity and artemisinin for its antimalarial properties [8,9]. Natural products and their derivatives dominate among drugs used to treat CNS diseases [10,11,12,13].

Terpenes constitute the largest cluster of secondary plant metabolites, encompassing a plethora of over 50,000 distinct substances, each characterized by a diverse array of biological attributes [14]. The most widespread terpenes are monoterpenes consisting of two isoprene fragments [15]. Monoterpenes that incorporate heteroatoms, such as oxygen, are categorized as monoterpenoids. As natural products, monoterpenes and monoterpenoids are the subject of increased attention from the world scientific community in the search for new pharmacological agents in various branches of medicine and pharmacy [16,17,18,19,20,21,22,23,24,25,26]. They have many biological properties, including antifungal, antibacterial, antioxidant, anticancer, antispasmodic, hypotensive, vasodilating effects, etc. The review by Yang et al. (2020) on the biological properties of terpenoids addresses the topic of their therapeutic potential and confirms the interest of researchers in developing terpenoid derivatives with different pharmacological properties [27]. A recent study from 2022 (Kumar et al.) summarizes the current data about the therapeutic potential of terpenes and terpenoids, confirming that these natural substances are widely distributed in various plant and marine sources and possess a wide range in biological properties [28].

**Figure 1:**
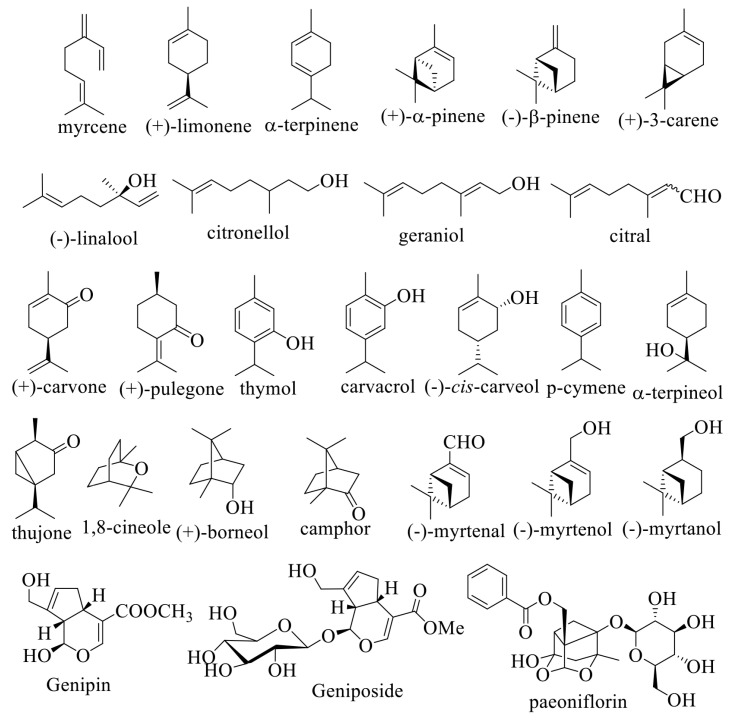
Some bioactive monoterpenes and monoterpenoids.

In vitro and in vivo studies reveal their potential therapeutic effects on diabetes, insulin resistance, and obesity, as described by Habtemariam (2017) [29] and Al Kury et al. (2021) [30]. Genipin and geniposide (Figure 1), along with iridoids and their glycosides, exhibit in vitro antidiabetic properties. Geniposide stimulates insulin secretion by activating the glucagon-like-1 (GLP-1) receptor [31]. Limonene showed a promising in vivo antidiabetic effect, established by Ramakrishnan and Ramalingam in 2012 [32]. Borneol, citronellol, and myrtenal increased liver glycogen levels. Carvacrol, carvone, citronellol, genposide, myrtenal, and paeoniflorin modulated key liver enzymes of glucose metabolism, reducing the activity of glucokinase and glucose-6-phosphate dehydrogenase.

In a clinical study from 2013, Luft et al. proved the role of chronic inflammation in the pathogenesis of diabetes and obesity [33]. Many of the monoterpenes exhibit positive effects on inflammatory conditions [34], also associated with diabetes. The in vitro study by Kong et al. (2013) reported that monoterpenes directly improve the lipid profile by affecting key enzymes for fatty acid synthesis (acetyl-CoA carboxylase and synthase) [35]. De Sousa (2011) summarized the available data about the analgesic potential of many plant essential oils that are rich in monoterpenes [36]. Numerous research works have been carried out in this important field in the last decade. High analgesic activity was demonstrated for (−)-linalool [37] and its acetate [38], citronellal [39], p-cymene [40,41], camphor [42], α-terpineol [43,44,45,46], menthol [47], myrcene [48], limonene [49,50], pulegone [51], citronellol [52], cuminic alcohol [53], and 1,8-cineole [54]. The anti-inflammatory activity of monoterpenes was reviewed by Araruna et al. (2020) [55].

In certain instances, positive outcomes have been observed as an adjunct to radiation therapy in the context of cancer treatment. They have the potential to be “radiosensitizers”, increasing the in vitro sensitivity of cancer cells (head and neck tumors) to radiation therapy with subsequent induction of apoptosis [56]. In addition to the studies on their cytotoxic properties as potential antitumor agents, research on their toxicity and potential risk of use is also of interest. Wojtunik-Kulesza (2022) revealed the toxic potential of α-terpinene, camphor, citral, limonene, pulegone, thujone (Figure 1), and the essential oils that contain them [57]. Genotoxicity, embryotoxicity, neurotoxicity, and allergenicity were indicated.

Memory-enhancing properties have also been reported for some of the studied monoterpenes [58]. For example, thymol’s positive effect on cognitive deficits, associated with a high-fat diet in mice, was found by Fang et al. (2017) [59]. According to Deng, Lu, and Teng (2013), carvacrol counteracted memory impairments in rats associated with experimental diabetes by affecting different mechanisms [60]. Some of the bicyclic monoterpenoids inhibit acetylcholinesterase activity, which is elevated in Alzheimer’s disease patients. In the Miyazawa and Yamafuji’s (2005) study of 17 representatives from this group of substances, (+)- and (−)-α-pinene and (+)-3-carene (Figure 1) appeared as moderate inhibitors of the enzyme, while bicyclic ketones and alcohols are weak anticholinesterase agents [61]. Their oxygenated derivatives and paramenthane monoterpenoids were found to be even less active.

**Figure 2:**
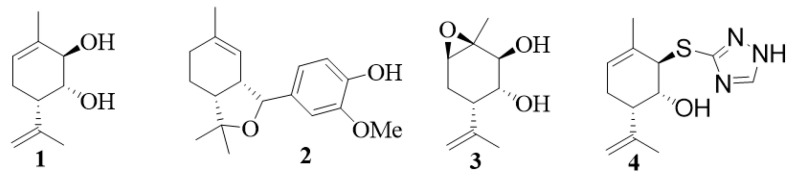
Some monoterpenoids with antiparkinsonian activity (**1–4**).

Several monoterpenoid alcohols, including geraniol [62,63], diol **1** derived from verbenol [64], and a conjugate **2** obtained by reaction of (+)-2-carene with vanillin [65], demonstrated promising antiparkinsonian activity in vivo in an MPTP (1-methyl-4-phenyl-1,2,3,6-tetrahydropyridine) mouse model. Derivatives of diol **1**, compounds **3** and **4**, showed neuroprotective effects in vitro and in several experimental mice models (MPTP-, rotenone-, and haloperidol-induced) of Parkinson’s disease [66,67] (Figure 2). Neuroprotective potential was demonstrated also for carvacrol [68,69,70], limonene [71,72,73], geraniol [74,75], citronellol [76], carveol [77], 1,8-cineole [78], paeoniflorin [79], α-pinene [80], and linalool [81].

Guzmán-Gutiérrez et al. (2012) reported an antidepressant effect of β-pinene and linalool [82], and Deng et al. (2015) found that thymol demonstrated this property in a model of chronic mild stress in mice [83]. Several monoterpenoids have demonstrated anxiolytic effects in rodents [84]. In 2015, de Sousa et al. published a systematic review of the anxiolytic-like effect of essential oils and their components, mainly monoterpenoids [85].

The advancement of novel prospective medications within the domain of monoterpene derivatives represents a promising approach to the treatment of various diseases, signifying a progressive trajectory in the field of experimental pharmacology.

## 2. Therapeutic Potential of Monoterpenoid Derivatives

A scarcity of substantiated data exists regarding the positive effects of monoterpene derivatives. Shi et al. (2016) reported on the antiallergic properties of some peony monoterpenes derivatives [86]. The review by Salakhutdinov, Volcho, and Yarovaya (2017) summarized the currently available data on the presence of various types of biological activity exhibited by monoterpenes and their derivatives. Their analgesic, anti-inflammatory, anticonvulsant, antidepressant, anti-Alzheimer’s, antiparkinsonian, antiviral, and antibacterial (antituberculosis) properties were described [87]. In addition to these data, a compound synthesized by the interaction of (−)-myrtenal and 2-aminoadamatane was found to have anxiolytic activity in Elevated plus maze test in mice [88]. In 2020, Zielińska-Błajet and Feder-Kubis provided a comprehensive summary of recent advancements in using derivatives of borneol, camphor, geraniol, myrtenal, pinene, and thymol as biologically active substances [89]. In 2021, Silva et al. reviewed the potential of 16 monoterpenes and their derivatives to affect various models of cardiovascular disease [90]. According to Bergman, Franks, and Philips (2023), certain acyclic monoterpenes and their derivatives have demonstrated notable anti-inflammatory potential [91]. Several monoterpene–coumarin conjugates showed good antiviral activity [92,93], while others demonstrated the capacity to inhibit the enzyme TDP1, which serves as a pivotal target in the realm of anticancer therapy [94,95]. The same inhibitory activity was demonstrated for some monoterpenes conjugated with various heterocyclic fragments [96,97]. Cardoso et al. (2021) explored some monoterpene derivatives as P-glycoprotein inhibitory activity agents in cancer cell resistance [98]. Tree monoterpene alkaloid hydrazone derivatives studied by Paterna et al. (2015) showed apoptosis-inducing properties in human colon and liver carcinoma cells [99].

Therefore, chemical modification of natural substances by conjugation with various synthetic components is a modern method for obtaining new biologically active compounds. It had been established that in many cases the medicinal properties of the obtained derivatives are more pronounced than those of the parent substances, and may even exceed the effects of the standards used in various therapeutic areas. It is necessary to carry out more in-depth and extended studies of such compounds. In view of their sufficient safety profiles, these compounds could subsequently be included in a range of clinical studies.

## 3. Therapeutic Potential of Myrtenal

**Figure 3:**
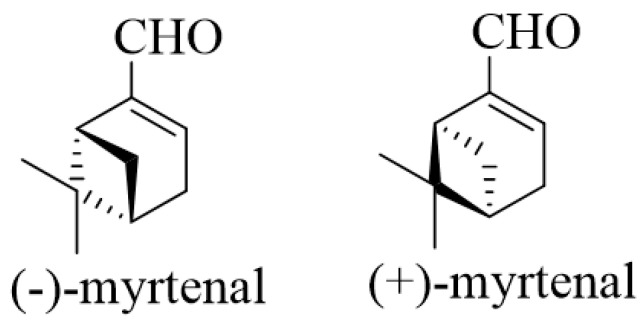
Structure of (−) and (+)-Myrtenal.

(−)-Myrtenal, (1*R*)-2-pinen-10-al, (1*R*)-6,6-dimethylbicyclo[3.1.1]hept-2-en-2-carboxaldehyde (Figure 3), is a bicyclic monoterpenoid of natural origin.

Myrtenal is considered practically insoluble in water and relatively neutral. Data on its mammalian metabolism are limited. In plants, myrtenal is metabolically related to α-pinene [100] because α-pinene is metabolized to myrtenol with subsequent transformation to myrtenal [101]. The major metabolite of the monoterpenoid in rabbits is myrtenic acid [102]. Furthermore, according to Scheline (1991), the structural similarity between alpha-pinene and myrtenal and the formation of common metabolites is the reason to assume that M is a transition compound in pinene metabolism [103].

The toxicological characterization of myrtenal is incomplete and provides information only on the lack of genotoxicity (EFSA FAF Panel, 2019) [104].

The substance is a component in the essential oils of many plant species [105] (Dragomanova et al., 2018). It is naturally present in mandarin peel oil, raspberry, blackberry, strawberry, ginger, hop oil, black tea, peppermint oil, pepper, myrtle leaf or berry, and summer savory, along with other spices or foodstuffs including hyssops, rosemary, spearmint, and many more. Table 1 presents the sources with the highest myrtenal content in their essential oils.

Medicinal plants containing myrtenal in their essential oils possess a wide range of biological properties. In the 20th century, bronchodilator, anti-inflammatory, antiaggregative, antihemolytic (in vitro), and antibacterial (against G (+) pathogens) effects of myrtenal were discovered in experimental animals [122]. This explains the use of plant essential oils containing myrtenal in aromatherapy for infections of the upper respiratory tract. They have the potential to favorably affect various systems and organs, including CNS functions, which is discussed in the review by Dragomanova et al. (2018) [105].

According to Saito et al. (1996) [123] and Santos et al. (2011) [124], the compound induced vasodilation, decreased heart rate, and improved hypotension in doses 1 and 5 mg/kg b.wt. after i.v. application in laboratory rats. Many other biological properties of myrtenal have been established.

### 3.1. Antidiabetic Potential

**Figure 4:**
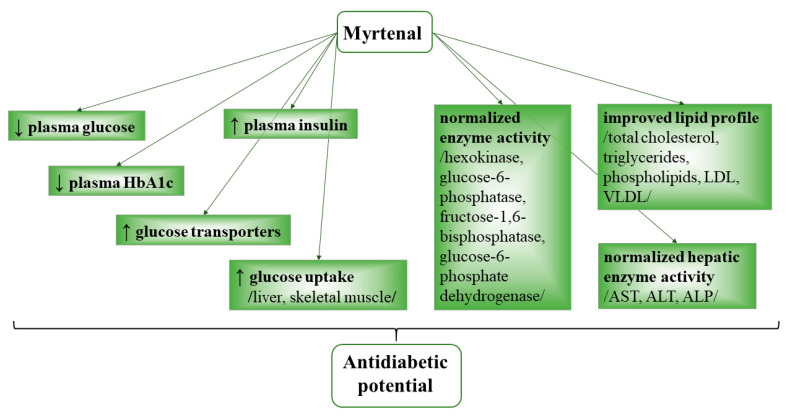
Established mechanisms of antidiabetic activity of myrtenal.

Myrtenal exhibited an antihyperglycemic effect in rats with an experimental streptozotocin-induced diabetes mellitus model. The compound lowered plasma glucose levels, improved plasma insulin levels, upregulated various glucose transporters, and subsequently improved glucose uptake in the liver and skeletal muscle. In the study by Rathinam and Pari (2016) [125], oral administration of myrtenal (80 mg/kg b.wt.) for 28 days produced a number of effects in rats with induced diabetes. It reduced plasma glucose and glycated hemoglobin A1c (HbA1c); increased levels of insulin and hemoglobin; regained body weight; and normalized activity of hexokinase, glucose-6-phosphatase, fructose-1,6-bisphosphatase, glucose-6-phosphate dehydrogenase and liver enzymes AST, ALT, and ALP. Myrtenal also increased glycogen content in the liver and muscles; recovered the hepatocytes; and improved pancreatic insulin levels and lipid profile values (total cholesterol, triglycerides, phospholipids, low-density lipoprotein, very-low-density lipoprotein, atherogenic index) (Figure 4).

### 3.2. Antitumor Potential

**Figure 5:**
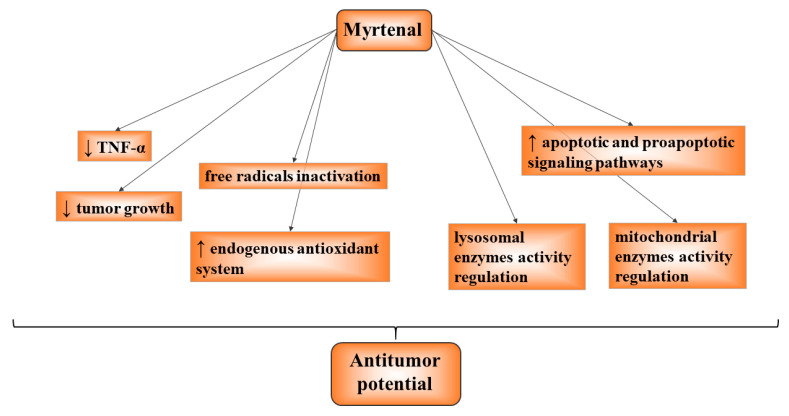
Established mechanisms of antitumor activity of myrtenal.

Myrtenal has been shown to have antioxidant and antitumor activity in peroral administration at a dose of 230 mg/kg b.wt. in corn oil for 28 days. These effects were achieved through a variety of mechanisms of action, including stabilizing endogenous antioxidant protection, influencing apoptotic and proapoptotic signaling pathways, inhibiting the expression of TNF- and reducing tumor growth, and regulating the activity of several lysosomal and mitochondrial enzymes [126,127,128,129]. The monoterpenoid was administered for 28 days in the experimental protocols of Babu et al., and Venkatachalam’s research group applied it for 15 weeks. Myrtenal was found to inhibit V-type ATPase on the surface of tumor cells in an in vitro study in melanoma cell lines, leading to their death and also to the suppression of melanoma metastasis in experimental mice at a dose of 15 mg/kg b.wt. (i.p. application for 21 days) [130]. The monoterpenoid demonstrated the ability to inactivate free radicals, resulting in inhibition of colon carcinogenesis and suppression of tumor progression after 30 weeks of intragastric administration at a dose of 230 mg/kg b.wt. [131,132]. Trytek et al. (2018) investigated the antitumor potential of (−)-myrtenol, (−)-myrtanol, and (−)-myrtenal (Figure 1) on human colon carcinoma cells in vitro and found that myrtenal had the highest activity [133]. The proposed mechanism of antitumor characteristics was that these substances affect the mitochondrial enzymes’ activity and membrane stability (Figure 5).

Our results confirmed the antioxidant mechanisms in the CNS effect of myrtenal (40 mg/kg b.wt. applied intraperitoneally for 11 days) in intact rats, in the brain of which myrtenal caused SOD (superoxide dismutase) activity and malondialdehyde (MDA) levels to decrease, and total glutathione (tGSH) content to increase, compared to controls [134].

### 3.3. Analgesic Potential

Pinene, whose derivative is M, showed analgesic potential in various models of induced pain [135]. On the other hand, the hydroxyl derivative myrtenol suppressed nociceptive and inflammatory responses in experimental conditions by inhibiting cell migration and neuromediation in pain pathways [136]. Intraperitoneal administration of this alcohol in experimental mice reduced the number of spasms in the acetic acid writhing test. For the first time, a recent investigation demonstrated myrtenal’s analgesic potential in laboratory mice. After a single, 7-day, and 14-day i.p. administration (30 mg/kg), the analgesic effect of M was established in two pain models: the acetic acid writhing test (antipyretic type analgesia) and the hot plate test (narcotic-type analgesia) [134].

### 3.4. Anti-Inflammatory Potential

The anti-inflammatory activity of *Myrtus communis*, L. extracts was established in two chronic inflammation mice models: a xylene-induced ear edema and a cotton pellet test [137]. In rats with induced rheumatoid arthritis, myrtenal isolated from *Liquidambra formosana* L. exhibited anti-inflammatory properties [138]. They were manifested by lowering the plasma levels of interleukin-1β (IL-1β) and tumor necrosis factor α (TNF-α), and also by suppressing the activation of the nucleotide-binding, oligomerization domain (NOD)-like receptor family, pyrin domain containing 3 (NLRP3) inflammasome, which was confirmed in vitro.

### 3.5. CNS-Affecting Potential

Subsequent research into the myrtenal effects on the CNS in our experiments on laboratory rodents showed potentiation of the classical sedative–hypnotic action of the drugs. In our opinion, these results are due to the interaction of myrtenal (20 and 30 mg/kg i.p. in a single dose) with the GABA receptor, since the introduction of the benzodiazepine antagonist Flumazenil (0.5 mg/kg) is followed by a sharp recovery in the condition of the experimental animals [134]. The central mechanism for action of myrtenal and its influence on GABA-ergic neurotransmission is related to the established anxiolytic properties of the substance. Hailu et al. (2011) reported substantial anxiolytic effects of myrtle essential oil, which were found to be comparable to those of diazepam [139]. Our studies in mice also confirmed the anxiolytic property of myrtenal according to the marble-burying test after single and repeated 7- and 14-day i.p. administration at 30 mg/kg dose, comparable to that of diazepam (1 mg/kg) as a reference [134].

### 3.6. Neuroprotective Potential

The neuroprotective potential of myrtenal was investigated in an injury model in which experimental mice were exposed to radiofrequency electromagnetic radiation during the gestational and neonatal periods. Akefe et al. (2023) administered myrtenal orally at doses of 100 and 200 mg/kg for 28 days, observing improvement in memory processes and in some biochemical parameters [140]. The substance improved short-term memory and spatial orientation. Additionally, it restored the activity of antioxidant enzymes, thereby rectifying the oxidative–inflammatory status within the brains of the experimental mice. The restoration of cholinergic neurotransmission and the levels of dopamine, noradrenaline, and serotonin manifested myrtenal’s neuromodulatory properties.

**Figure 6:**
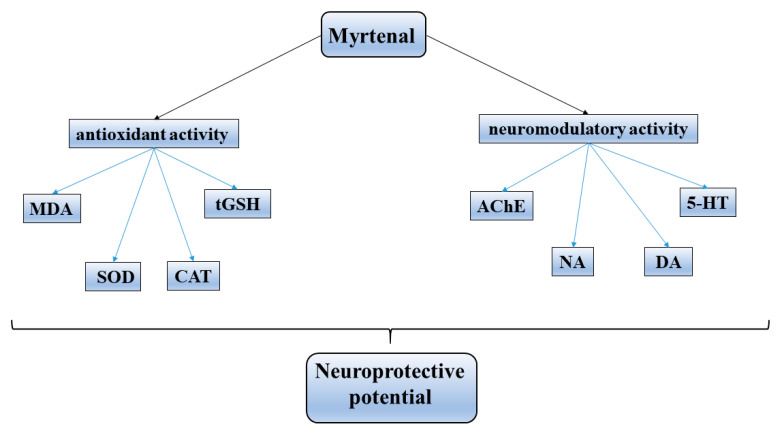
Established mechanisms of neuroprotective activity of myrtenal.

The ability of natural myrtenal to affect neurodegenerative diseases was demonstrated for the first time by our team. In two experimental models of neurodegeneration—6-OHDA-induced parkinsonism [141] and chemically induced dementia [142] in rats—we established the antioxidant potential of the compound (30 mg/kg i.p. for 5 days), as an element of its neuroprotective activity. There are limited data in the literature on myrtenal’s effects on the levels of major brain neurotransmitters. In the report by Kaufmann, Dogra, and Wink (2011) [143], in vitro anticholinesterase effects were not confirmed by the presence of direct inhibition of enzyme activity in our in vivo experiments in mice (20 mg/kg, i.p. for 11 days) [134] and rats (40 mg/kg, i.p. for 9 days) with experimental neurodegeneration [143]. We established a significant protective effect of myrtenal on neurodegenerative processes in experimental rodent models [140]. Under the conditions of our studies in a scopolamine injury model of dementia, the monoterpenoid exhibited neuromodulatory properties through a slight nonsignificant decrease in brain AChE activity, a significant decrease in hippocampal noradrenaline content, and increased hippocampal and cortical serotonin levels in experimental rats. Examining the myrtenal (50 mg/kg i.p. for 5 days) neuroprotective effects on an experimental 6-OHDA model of Parkinson’s disease further revealed the multitarget mechanism of its action. It demonstrated positive effects on the memory and learning abilities and on the coordination and exploratory behavior of experimental rats, via reducing the main parameters of brain oxidative stress, and increasing the dopamine content in the damaged cerebral hemisphere [141]. According to these first studies, we demonstrated at least two mechanisms of action that are involved in the protective effects of myrtenal on neurodegenerative processes: neuromodulatory and antioxidant (Figure 6).

Although many biological properties of myrtenal have been established, no therapeutic agents have yet been developed. Currently, only one pharmaceutical product based on natural myrtenal is available, Myrtecaine, and it has a local anesthetic effect. Its combination with diethyl amine and salicylic acid is used for muscle and joint pain topical treatment. On the other hand, there is increasing interest in its derivatives and their therapeutic potential in various fields of the medical practice.

## 4. Therapeutic Potential of Myrtenal Derivatives

Monoterpenes and their derivatives serve as pivotal starting components in the design and synthesis of novel biologically active compounds.

### 4.1. Antitumor Potential

**Figure 7:**
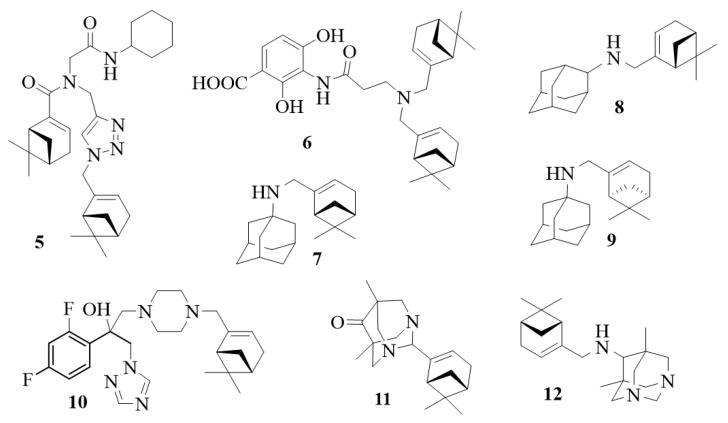
Structures of biologically active myrtenal-derived compounds (**5**–**12**).

Concepción et al. (2020) described several basic structures of myrtenal conjugates such as compound **5** (Figure 7) with high cytotoxicity against cancer cells with EC_50_ in the nanomolar range [144]. These novel myrtenal-conjugated pseudo-peptides have been synthesized and investigated as prospective antitumor agents. They represent potential candidates for anticancer therapy due to their selective cytotoxic activity. Some of these compounds exhibited notable in vitro cytotoxicity against human gastric, breast, and colon adenocarcinoma cell lines, while demonstrating no such effect against human dermal fibroblast cell lines. One derivative exhibited acceptable EC_50_ and Emax values in cancer cell lines and in inducing cytotoxicity in actively proliferating CD4+—T cells without affecting nonproliferating T cells.

Zielińska-Błajet and Feder-Kubis (2020) provided an overview of diverse therapeutic effects on selected aliphatic, monocyclic, and bicyclic monoterpenes such as geraniol, thymol, myrtenal, pinene, camphor, borneol, and their modified structures [89]. A recent study with fourteen newly synthesized perillaldehyde and myrtenal-based benzohydrazides revealed the antiproliferative potential for two of them [145]. Increasingly more literature data are available regarding new modifications of these natural compounds, including their biological effects and medical applications. However, information regarding the biological and pharmacological properties of monoterpene derivatives remains limited to several review articles, published in the last ten years [91,122,146,147,148,149,150,151,152].

In 2011, Wang and Sintim developed compound **6** (Figure 7), a myrtenal analog of natural antibiotics (platensimycin, platencina, and (−)-myrtamycin) with promising antibacterial activity at MIC 4 µg/mL [153].

In 2015, Suslov et al. published that amino adamantane derivatives of myrtenal **7** and **8** possess potent cytotoxic effects against the tumor lines used (CTD_50_ = 12÷21 μM), along with low toxicity with respect to MDCK cells [154]. Compound **7**, which was synthesized from 1-amino adamantane and (–)-myrtenal, showed cytotoxic activity against CEM-13, MT-4, and U-937 human cancer cells. Adamantylamine derivative demonstrated high activity against all tumor lines used, along with low cellular toxicity, while the 1-amino adamantane and (+)-myrtenal-derived compound **9** exhibited antitumor potential by inhibiting tyrosyl-DNA phosphodiesterase 1 (TDP1) activity (IC_50_ 6 μM), an important target for antitumor therapy, according to research by Ponomarev et al. (2018) [155]. These myrtenal derivatives do not exert genotoxic properties. An even more active TDP1 inhibitor with IC_50_ 45 nM was developed by combining myrtenal and usnic acid fragments [156].

Gonda and Szakonyi (2018) reported the synthesis of 1,2,4- and 1,3,4-oxadiazole derivatives of (–)-myrtenal [157]. All compounds were tested in vitro for antiproliferative activity against four human malignant cell lines using the MTT [3-(4,5-dimethylthiazol-2-yl)-2,5-diphenyltetrazolium bromide] assay [158]. One of them inhibited tumor growth, with IC_50_ values comparable to those of the reference cisplatin, but showed lower antiproliferative activity against the triple-negative breast cancer cell line (MDA-MB-231) compared to other cell lines used in gynecology. The remaining compounds exhibited a relatively diminished level of activity against ovarian cancer (cell line A2780).

### 4.2. Anxiolytic Potential

Kapitsa et al. (2012) described new nitrogen-containing compounds with an adamantane–myrtenal structure and then investigated the anxiolytic activity of the resulting products in male Balb/C mice by using the elevated plus maze test [88]. The results showed that compound **8** exhibited anxiolytic potential upon single administration.

### 4.3. Antiviral Potential

Teplov et al. (2013) tested in vitro the same conjugate of 2-amino adamantane and (–)-myrtenal for antiviral activity against influenza virus A/California/07/09 (H1N1)pdm09 and found that the introduction of a myrtenal fragment led to an increase in the antiviral activity of the adamantylamine derivatives against the adamantylamine-resistant virus [159]. The selectivity for most of the synthesized amines surpassed that for Rimantadine and Amantadine.

### 4.4. Antifungal Potential

Compound **10**, which is a myrtenol-containing analogue of azole antifungals, demonstrated promising antifungal activity against both fluconazole-susceptible and fluconazole-resistant strains, including fluconazole-resistant clinical isolates of Candida parapsilosis and Candida glabrata, with excellent minimum inhibitory concentration in submicrogram and nanogram range. The compound was up to 100 times more active than fluconazole [160].

### 4.5. Analgesic Potential

A high analgesic effect with an active dose of 20 mg/kg was shown for myrtenal-derived diazaadamantanone **11** [161]. The compound has a low acute oral toxicity with LD_50_ of more than 1000 mg/kg and does not cause damage to the gastric mucosa. Similar analgesic activity was demonstrated for diazaadamantane–myrtenal conjugate **12** [162]. Having in mind evidences for analgesic potential of myrtenal established in our previous studies, it can be concluded that this analgesic potential can be improved and extended via synthesis of some diazaadamantanone analogues of myrtenal.

### 4.6. Memory-Improving Potential

The conjugates of amino adamantane with myrtenal **7** and **8**, in the form of hydrochlorides, studied by Kapitsa (2012) [88] and Teplov (2013) [159], showed the potential to influence memory processes in intact rats after 11 days of repeated intraperitoneal administration at a dose of 1 mg/kg [163]. The two compounds of amino adamantane with myrtenal investigated in this study exhibited antiacetylcholinesterase activity. Additionally, they demonstrated the capability to influence the levels of norepinephrine and serotonin in the cerebral cortex and hippocampus of the experimental animals. Notably, one of these substances also displayed antidepressant potential, which can be attributed to the induced increase in brain monoamines concentrations, the reduced levels of which are associated with depressive conditions.

Our pilot study of these two amino adamantane compounds with myrtenal was extended by examining their effects on scopolamine-induced neurodegeneration in rats [164]. The derivatives again caused AChE activity inhibition in the cerebral cortex of dement rats, accompanied by some antioxidant capacity expressed by increased glutathione content. They also affected the levels of norepinephrine and serotonin in the cortex and hippocampus of laboratory rodents in an induced model of dementia, as was observed in intact rats in the previous study. These studies, for the first time, reveal the capacity of two amino adamantine conjugates with myrtenal to influence neurodegenerative processes through different mechanisms of action: antiacetylcholinesterase and neuromodulatory.

## 5. Future Perspectives

**Figure 8:**
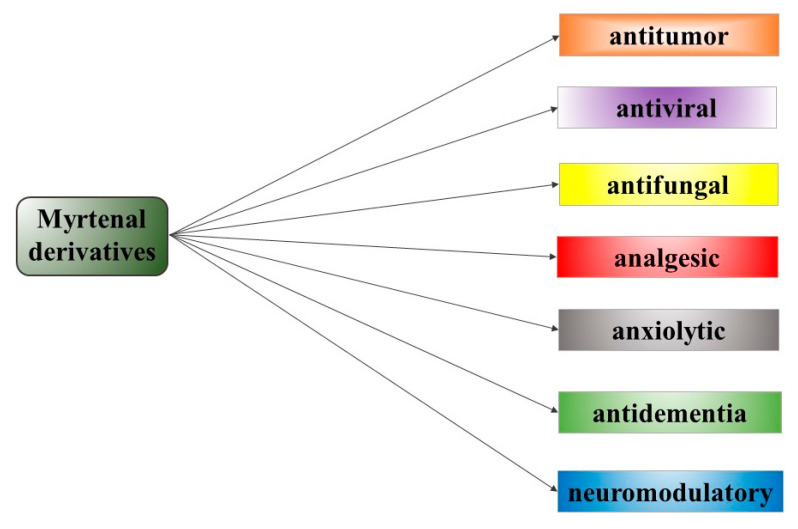
Therapeutic potentials of myrtenal derivatives.

Myrtenal derivatives showed a broad spectrum of activities, which reveal myrtenal’s potential as a basis for the design of new pharmacological agents (Figure 8).

Some of the compounds showed new activities different from those of myrtenal, while others showed an enhancement of the effects of the natural substance. Different combinations of myrtenal and adamantane moieties have gained interest in various fields, including medical chemistry and drug discovery. Some research focuses on the design, synthesis, and evaluation of myrtenal–adamantane conjugates as potential pharmacological agents. Moreover, other analogs of these conjugates with modification of monoterpene, adamantine, and linker structures could be considered in the future. This group of compounds showcased a spectrum of beneficial properties that warrant comprehensive and in-depth examination. They have the potential to be introduced in the future into various areas of the pharmacotherapeutic practice. Several possible areas of applications of these derivatives include antimicrobial (antifungal and antiviral), anticancer, anxiolytic, and neuroprotective activity.

## 6. Conclusions

As a natural compound with a wide range in biological activities, myrtenal can have future implementation in medical practice. The chemical modification of this monoterpenoid with some pharmacophores will allow the enhancement of its essential properties, thereby augmenting its therapeutic potential.

## Figures and Tables

**Table 1:** Myrtenal-containing medicinal plants and other natural sources.

Natural Source	Plant Part	Reference
*Artemisia* spp.	leaves, stems, and flowers/fruits	[106]
*Coriandrum sativum*	seed oil	[107]
*Cuminum cyminum*	essential oil	[108,109]
*Curcuma amada, Curcuma aromatica*	essential oil	[110]
*Glycyrrhiza glabra*	root essential oil	[111]
*Helianthus annuus*	flower heads	[112]
*Hyssopus officinalis*	essential oil	[113]
*Juglans regia*	leaf essential oil	[114]
*Laurus nobilis*	leaves	[111]
*Lavandula* spp.	essential oil	[115]
*Ledum palustre*	essential oil	[116]
*Myrtus communis*	essential oil	[117]
*Origanum majorana*, *Origanum vulgare*	essential oil	[118]
*Peumus boldus*	flower	[111]
*Piper nigrum*	fruit	[111]
*Propolis*	-	[119]
*Rosmarinus officinalis*	essential oil	[120]
*Thymus* spp.	essential oil	[121]

## Data Availability

Not applicable.
